# Albumin for sepsis-related peripheral tissue hypoperfusion

**DOI:** 10.1186/s13054-024-04846-x

**Published:** 2024-03-14

**Authors:** Mingqiang Wang, Lin Zhong

**Affiliations:** 1 Department of Critical Care Medicine, Zhengzhou University People’s Hospital, Henan University People’s Hospital, Zhengzhou, China; 2 Henan Key Laboratory for Critical Care Medicine, Zhengzhou Key Laboratory for Critical Care Medicine, Henan Provincial People’s Hospital, Zhengzhou, China; 3 Department of Critical Care Medicine, The First People’s Hospital of Pinghu, Pinghu, China

We read with interest the manuscript by Gabarre et al. regarding albumin infusion [1]. Here, I believe there is a minor issue that needs clarification.

The sample size in each group of this study is quite small. On this basis, there is a difference in the need for vasopressor between the two groups, although it did not reach statistical significance (0.4 vs. 0.7, *P* = 0.06). Considering that all patients received 20 mg/kg of saline, could this lead clinicians to perceive poorer response to saline in this subset of patients, prompting additional selection of albumin?

In our clinical practice, we frequently supplement albumin instead of crystalloids for patients who exhibit a poor response to crystalloids therapy [2]. It is challenging to precisely define what constitutes a poor response to crystalloids, but often our intuition, along with hourly assessments showing non-decrease in vasopressor requirements after continuous crystalloids infusion, informs us that these patients may no longer benefit from saline and that albumin could be a viable alternative.

Therefore, it cannot be simply assumed that “The decision to administer saline or albumin, left to the discretion of the attending physician, may pose a confounding factor.” Instead, it is possible that clinicians, recognizing a poor response to saline in certain patients, with a slow reduction in vasopressor usage, chose to administer albumin as an alternative.

Performing further analysis after Inverse Probability of Treatment Weighting for vasopressor usage or conducting a well-designed RCT might yield more accurate conclusions.

## Data Availability

None.
